# Chondrosarcome myxoïde extra-osseux: à propos d'un cas et revue de la littérature

**DOI:** 10.11604/pamj.2015.20.360.6384

**Published:** 2015-04-14

**Authors:** Youness Sasbou, Abdelkarim Rhanim, Younes Mhammdi, Mustapha Nkaoui, Ahmed El Bardouni, Mohammed Saleh Berrada, Morad El Yaacoubi

**Affiliations:** 1 Service de Traumatologie-Orthopédie, CHU Ibn Sina, Rabat, Maroc

**Keywords:** Chondrosarcome, extra osseux, parties molles, myxoïde, membres, Chondrosarcoma, extra skeletal, soft tissue, myxoid, limbs

## Abstract

Le chondrosarcome primitif des tissus mous est beaucoup plus rare que son homonyme intra-osseux, le type myxoïde est une entité distincte sur le plan clinique, histologique, immuno-histo-chimique, cytogénétique et évolutif. Le but de cette étude est d’évaluer les caractéristiques de cette tumeur et de mettre en évidence sa difficulté diagnostique et thérapeutique. Cette étude rapporte un cas de chondrosarcome myxoïde extra-osseux atteignant les parties molles, il s'agit d'une femme âgée de 28 ans ayant une localisation au niveau de la face antéro-externe du tiers moyen de la cuisse gauche, la patiente a été explorée par une IRM. L'examen histologique a confirmé le diagnostic et la patiente a bénéficié d'une résection large complétée par une chimiothérapie adjuvante, et le suivi n'a déploré aucune récidive ni métastase. Le diagnostic des chondrosarcomes primitifs extra osseux est particulièrement histologique et leur traitement repose sur la résection chirurgicale large suivie d'une chimiothérapie adjuvante.

## Introduction

Le chondrosarcome myxoïde extra-squelettique est une tumeur maligne rare décrite pour la première fois en 1953 par Stout et Verner [1]. Elle représente moins de 3% des sarcomes des tissus mous [2]. Appelée également sarcome chordoïde, elle est actuellement classée parmi les tumeurs à différenciation incertaine, en raison du manque d'arguments probants en faveur d'une différenciation cartilagineuse. Son pronostic est sombre, jalonné de récidives locales et de métastases principalement pulmonaires. Cette observation nous permet de rappeler les caractéristiques de cette tumeur en insistant sur la difficulté du diagnostic surtout anatomo-pathologique.

## Patient et observation

**Figure 1:**
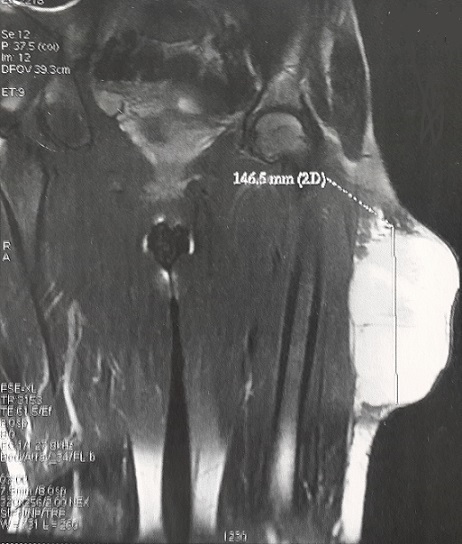
IRM en coupe coronale en séquence pondérée T2 montrant une masse tumorale assez bien limitée en hypersignal

**Figure 2:**
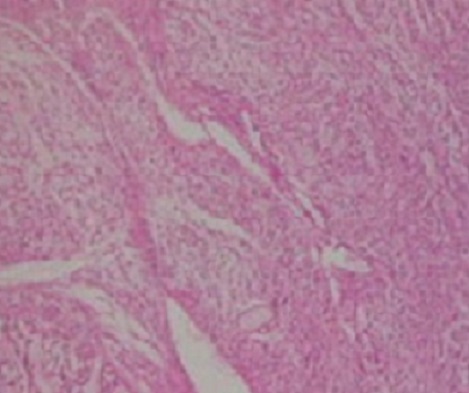
Architecture lobulée, fond myxoïde, cellules atypiques dans des logettes

**Figure 3:**
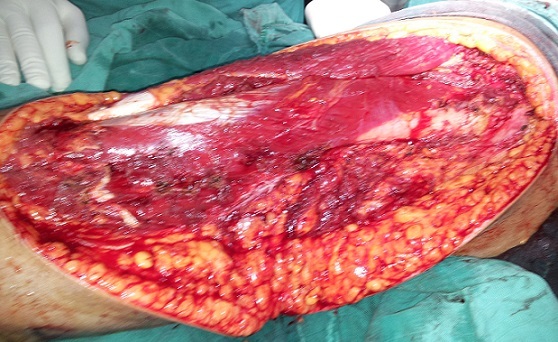
Vue per-opératoire montrant la résection carcinologique de la tumeur

Il s'agit de Mme N.F, âgée de 28 ans et sans antécédents pathologiques particuliers, qui a consulté pour une tuméfaction sous cutanée, de la face externe du tiers supérieur de la cuisse gauche, de 10 cm de diamètre environ, évoluant depuis 1 année et ayant augmenté de volume depuis cinq semaines environ. L'examen IRM du membre inférieur montrait une masse tumorale assez bien limitée de 8X13X14,6 cm, siégeant en sous cutanée au niveau de la face externe du tiers supérieur de la cuisse gauche (Figure 1), multi loculée, de signal hyper intense en T1, hypo intense en T2, discrètement hétérogène, encapsulée sans extension osseuse ni intra- musculaire. Le bilan d'extension générale était négatif. Une biopsie chirurgicale a été réalisée. L'examen histologique montrait une prolifération tumorale d'architecture nodulaire délimitée par des septas fibro-vasculaires qui abritent des cellules à cytoplasme abondant éosinophile avec des noyaux ronds chromatiques, ces cellules se disposent en cordons et en pseudo acini en périphérie de ces nodules (Figure 2). Donc l'analyse histologique a conclu à un chondrosarcome myxoïde extra-squelettique. La patiente a bénéficié d'une exérèse complète de la tumeur (Figure 3), suivie d'une poly-chimiothérapie de 6 semaines. A 12 mois de recul, on ne notait pas de récidive locale ni de métastases à distance.

## Discussion

Le CME est une entité rare distincte sur le plan clinique, histologique, immuno-histochimique, cytogénétique et évolutif. Sur le plan clinique, elle survient chez le sujet adulte vers l’âge de 50 ans avec une prédominance masculine et un sex-ratio de 2/1 [3]. Les membres représentent la localisation la plus fréquente (80%); son siège est en fait ubiquitaire, mais une localisation au niveau de la cuisse et du genou doit attirer l'attention [4]. Les autres sites possibles sont: les doigts, intracrânien, rétro-péritoine, plèvre et os [5]. Les signes cliniques ne sont pas spécifiques; souvent, il s'agit de douleurs ou d'une tuméfaction qui peut ulcérer la peau. Les images radiologiques ne sont pas spécifiques: un aspect pluri-nodulaire et un hyper signal en T2 sont observés à l'IRM. Macroscopiquement, elle se présente sous la forme d'une masse bien limitée par une capsule fibreuse, ferme, homogène, grise ou gélatineuse à la coupe. La taille moyenne rapportée est de 7 cm (1-25 cm). L'aspect histo-pathologique usuel est celui d'une tumeur d'architecture pluri-nodulaire, faite de massifs, de lobules et/ou de travées. Les cellules tumorales sont monotones, de taille moyenne, le plus souvent ronde ou légèrement allongées, au cytoplasme clair ou éosinophile. Des inclusions rhabdoïdes sont observées dans 10% des cas. Le noyau est en général de petite taille, réniforme ou ovoïde, à chromatine fine, laissant apparaître un petit nucléole. Les atypies cyto-nucléaires et l'activité mitotique sont en général discrètes. Dans les formes typiques, les cellules sont dispersées dans une trame plus ou moins abondante, volontiers myxoïde. Des foyers de différenciation cartilagineuse sont exceptionnellement observés. La composante cellulaire peut être plus ou moins dense. Des variantes plus cellulaires sont décrites, avec des dispositions cellulaires en nappes (formes solides), mais aussi des formes pseudo-épithéliales et pseudo-glandulaires. Dans les formes pauci-cellulaires, les cellules peuvent être fusiformes, rappelant la morphologie des myo-fibroblastes. La matrice myxoïde est colorée par le bleu Alcian aux pH 2,5 et 1, coloration résistante à la hyaluronidase, suggérant la présence de sulfates chondroïtiniques. Les cellules tumorales expriment toujours la vimentine, dans environ un tiers des cas la protéine S 100 (marquage typiquement faible et focal), et souvent le CD 99. La cytokératine et l'EMA sont parfois exprimés de façon focale [1]. Les autres marqueurs ne sont pas exprimés, sauf parfois la NSE, la synaptophysine, plus rarement la chromogranine, suggérant une possible différenciation neuroendocrine [1, 2]. Le CMES est associé le plus souvent à une translocation chromosomique récurrente t(9;22) (q22;q12), conduisant à un réarrangement génétique entre le gène EWS, localisé sur le chromosome 22, et le gène TEC, localisé sur le chromosome 9 [1]. Le réarrangement peut être recherché par RT-PCR sur tissu fixé. D'autres translocations sont plus rarement observées [2]: t(9;15) (q22; q12), t(9;17) (q22;q11.2), t(9;17;15) (q22;q11;q22), t(2;13) (q32;p12), et t(11;22) (q11;p11). ur le plan évolutif, le CMES est caractérisé par une agressivité locale par rapport à leur homologue squelettique, avec des récidives locales assez fréquentes (50%). L’évolution est lente avec une survie prolongée et le pronostic à long terme est défavorable (30 à 70% de survie à dix ans) [3, 6]. Les métastases surviennent dans la moitié des cas, elles sont de localisations souvent pulmonaires, parfois au niveau des tissus mous, de l'os, des ganglions ou du cerveau. À l'heure actuelle, il n'y a pas de facteurs pronostiques bien établis, et ils sont pour la plupart sujet à controverse. Les principaux facteurs de mauvais pronostic rapportés sont la survenue chez l'homme, l’âge tardif de survenue, une taille tumorale supérieure à 10 cm, le siège proximal, le caractère incomplet de l'exérèse chirurgicale initiale, l'absence de réséquabilité et la découverte de métastases au moment du diagnostic [1, 6, 7]. Les critères histo-pronostiques tels que la nécrose, l'activité mitotique, le degré de différenciation ne semblent pas influencer le pronostic [8, 9]. Le traitement fait appel à la chirurgie première de la tumeur initiale et/ou des métastases, la radiothérapie et la chimiothérapie en première intention n'ayant pas fait leur preuve [10, 11]. Cette tumeur ne répond pas à la chimiothérapie et les résultats concernant la radiothérapie sont discordants.

## Conclusion

Le CMES est une entité rare distincte sur le plan clinique, histologique, immuno-histo-chimique, cytogénétique et évolutif. La clé diagnostique est morphologique, aidée par l'immuno-histochimie et l’étude génétique t (9;22). C'est une tumeur de diagnostic très difficile et souvent retardé, et malgré son agressivité surtout locale et la survie prolongée, elle est considérée comme un sarcome de bas grade de malignité ou de malignité intermédiaire.

## References

[1] Oliveira AM, Sebo TJ, Mc Grogy JE, Gaffey TA, Rock MG, Nascimento AG (2000). Extraskeletal myxoid chondrosarcoma: a clinicopathologic, immunohistochemical and ploidy analysis of 23 cases. Mod Pathol..

[2] Okamoto S, Hisaoka M, Ishida T (2001). Extraskeletal myxoid chondrosarcoma: a clinicopathologic, immunohistochemical, and molecular analysis of 18 cases. Hum Pathol..

[3] McGrory JE, Rock MG, Nascimento AG (2001). Extraskeletal myxoid chondrosarcoma. Clin Orthop..

[4] Kempson RL, Fletcher CDM, Evans HL (2001). Extraskeletal myxoid chondrosarcoma. Tumours of soft tissue..

[5] Mills SE, Gaffey MJ, Frierson HF (2000). Tumours of the upper aerodigestive tract and ear. AFIP Atlas of tumour pathology.

[6] Lucas DR, Heim S, Fletcher CDM, Unni KK, Mertens F (2002). Extra skeletal myxoid chondrosarcoma. Tumours of soft tissue and bone.

[7] Mizuho Y, Junichi I, Mamoru T (2007). Chondrosarcoma of the temporal bone. Auris Nasus Larynx..

[8] Meis-Kindblom JM, Bergh P, Gunterberg B (1999). Extraskeletal myxoid chondrosarcoma: a reappraisal of its morphologic spectrum and pronostic factors based on 117 cases. Am J Surg Pathol..

[9] Santacruz MR, Proctor L, Thomas DB, Gehrig PA (2005). Extraskeletal myxoid chondrosarcoma: a report of a gynecologic case. Gynecol Oncol..

[10] Algros MP, Collonge-Rame MA, Bedgejian I (2003). Différenciation neuroectodermique des chondrosarcomes extrasquelettiques myxoïdes? un classique?. Ann Pathol.

[11] Acero J, Escrig M, Gimeno M, Montenegro T, Navarro-Vila C (2003). Extraskeletal myxoid chondrosarcoma of the infratemporal fossa: a case report. Int J Oral Maxillofac Surg..

